# Intelligent manufacturing policy, ESG performance, and total factor productivity: Evidence from China

**DOI:** 10.1371/journal.pone.0311369

**Published:** 2025-02-03

**Authors:** Haiyue Liu, Jianwu Ren

**Affiliations:** Institute of Chinese Financial Studies, Southwestern University of Finance and Economics, Chengdu, China; University of Malaya: Universiti Malaya, MALAYSIA

## Abstract

Under the global wave of intelligence, intelligent manufacturing has become a crucial means of transforming and upgrading China’s manufacturing industry. Accurate evaluation of the implementation effects of intelligent manufacturing industry policies is an urgent issue. This study uses the introduction of the “Made in China 2025” policy as a quasi-natural experiment and employs the difference-in-differences method to investigate the impact of intelligent manufacturing policies on firms’ total factor productivity (TFP) and its mechanisms. These results indicate that implementing intelligent manufacturing policies significantly enhances firms’ TFP. Mechanism analysis reveals that intelligent manufacturing policies can improve firms’ ESG performance by enhancing green technology innovation capabilities, increasing capital market attention, and reducing internal control costs, thereby enhancing firms’ TFP. Heterogeneity analysis finds that intelligent manufacturing policies have a more pronounced effect on promoting TFP in large-scale enterprises, labor-intensive enterprises, firms with higher technical employee levels, companies in highly competitive industries, and enterprises in regions with higher levels of digital infrastructure development and lower economic development as compared to their counterparts. This study provides evidence of how intelligent manufacturing policies drive the high-quality and sustainable development of enterprises and offers insights for future policy formulation and implementation.

## Introduction

Since its reform and opening up, China has transformed from a structurally singular, weak manufacturing nation with a fragile foundation into a comprehensive and robust manufacturing giant. The added value of the manufacturing industry increased from 16.98 trillion yuan in 2012 to 33.5 trillion yuan in 2022, maintaining its position as the world’s largest manufacturing industry for 13 consecutive years. However, compared to developed countries, China’s manufacturing industry still faces key issues of being “large but not strong” and “comprehensive but not refined,” with weak technological innovation capabilities and a position at the lower end of the international trade value chain [1, 2]. Following the 2008 financial crisis, developed countries, led by the United States and Germany, initiated a wave of “re-industrialization.” Confronted with the dual challenges of foreign manufacturing reshoring and sluggish domestic growth momentum, China’s manufacturing sector must urgently transform its development model, optimize its industrial structure, and enhance its core competitiveness. Effectively improving firms’ independent innovation capabilities and production efficiency to advance the manufacturing industry toward the mid-to high-end is a pressing issue for China to address during its transition from a manufacturing giant to a manufacturing powerhouse.

In recent years, with the rapid advancement of emerging digital technologies, such as artificial intelligence, big data, blockchain, and cloud computing, intelligent manufacturing has increasingly become a critical strategic direction for global technological change. Intelligent manufacturing is a manufacturing model deeply integrated with next-generation information and communication technology and advanced manufacturing technology, encompassing various manufacturing stages such as design, production, and services. It not only has the potential to reshape traditional production models but also to create new production methods, and its application is bound to have a significant impact on business operations [3]. In the context of manufacturing transformation, China introduced an intelligent manufacturing development strategy aimed at fostering innovation in the manufacturing sector, enhancing resource allocation efficiency, and ultimately achieving high-quality sustainable development in manufacturing. However, research on whether intelligent manufacturing policies have achieved the objective of high-quality sustainable development in manufacturing is lacking. What impact do intelligent manufacturing policies have on firms’ total factor productivity (TFP)? Does implementing intelligent manufacturing policies promote firms’ ESG performance, thereby influencing their TFP? This study uses an intelligent manufacturing strategy as a quasi-natural experiment to assess the implementation effects of intelligent manufacturing policies. The research findings have considerable practical implications for advancing China’s manufacturing industry toward the higher end of the global value chain.

The existing literature on firm TFP has made notable advancements, with numerous scholars conducting in-depth analyses and evaluations from both micro and macro perspectives. Factors such as corporate governance [4], technological innovation [5], industrial policy [6], and international trade [7] significantly enhance firm TFP; whereas financing constraints [8], firm financialization [9], and economic policy uncertainty [10] inhibit firm TFP to some extent. Studies on the determinants of firms’ ESG performance have emerged more recently, primarily focusing on bank-firm interactions [11], corporate digitalization [12], executive incentives [13], capital market openness [14], ownership structure [15], corporate culture [16], and merger and acquisition activities [17]. Moreover, much of the research on the practical impact of intelligent manufacturing on business operations is still in the theoretical analysis and case study summary stages [18], lacking substantial empirical validation. While prior studies investigated the impact of ESG performance on firms’ TFP [19, 20], the interplay between intelligent manufacturing policies, firms’ ESG performance, and TFP has not been examined. Therefore, it is necessary to evaluate and discuss quantitatively whether the implementation of intelligent manufacturing policies can effectively enhance firms’ ESG performance and TFP.

Drawing on existing literature [21, 22], this study uses the introduction of the “Made in China 2025” policy as a quasi-natural experiment and utilizes a sample of listed manufacturing companies on China’s A-shares from 2010–2023 to study how the exogenous impact of intelligent manufacturing policies affects firms’ TFP. The study finds that compared to firms not affected by intelligent manufacturing policies, those influenced by such policies show a significant increase in TFP. Further discussion reveals that the implementation of intelligent manufacturing policies can enhance firms’ ESG performance by strengthening green technology innovation capabilities, increasing capital market attention, and reducing internal control costs, thereby leading to growth in firms’ TFP. The heterogeneity analysis indicates that the positive impact of intelligent manufacturing policies on TFP is more pronounced for larger firms, labor-intensive firms, firms with higher levels of employee technical skills, firms in more competitive industries, and firms located in regions with higher levels of digital infrastructure development and lower levels of economic development as compared to their counterparts.

Compared to existing literature, this study’s marginal contributions are mainly reflected in the following three aspects. First, from the perspective of firms’ TFP, it evaluates the microeconomic effects of the implementation of intelligent manufacturing policies, enriching the research on intelligent manufacturing and its economic impacts. Existing evaluations of the implementation effects of intelligent manufacturing policies are mostly confined to theoretical analyses, with only a few studies focusing on the short-term impacts of such policies, such as government subsidies and asset prices [21, 22], and lacking systematic assessments of the long-term effects. Based on a quasi-natural experiment on China’s intelligent manufacturing policy, this study accurately identifies the causal relationship between intelligent manufacturing and firms’ TFP using the DID method, providing a new perspective for understanding the microeconomic effects of intelligent manufacturing policies.

Second, this study adds new scientific evidence to the research on the factors affecting firms’ TFP. There is extensive research on the factors influencing firms’ TFP, with previous literature delving deeply into both macro and micro perspectives, such as the policy environment [6, 10], financial markets [23], firm behavior [7, 24], and firm characteristics [4, 25]. However, few studies have systematically explored the potential impacts of advancements in information technology, particularly the deep integration of intelligence and industrialization, on firms’ TFP. This study focuses on a distinctive influencing factor—the implementation of intelligent manufacturing policies—and, through empirical analysis, verifies the positive impact of these policies on firms’ TFP, thereby enriching the literature on the factors influencing firms’ TFP.

Third, by further analyzing TFP, this study empirically examines the role of intelligent manufacturing policies in promoting firms’ ESG performance. It also comprehensively investigates the channels through which intelligent manufacturing empowers firms to achieve high-quality sustainable development from three perspectives: green technology innovation capabilities, capital market attention, and internal control costs. A deeper understanding of the relationship between intelligent manufacturing policies and firm development is beneficial. Additionally, this study explores the heterogeneous effects of intelligent manufacturing policies on firms’ TFP in different internal and external environments, providing important references for government efforts to implement and promote intelligent manufacturing and enhance firms’ TFP.

The remainder of this paper is structured as follows: The “Literature review and research hypothesis” section reviews the literature and formulates the research hypotheses. The “Research design” section outlines the empirical research design. The “Empirical results” section presents the empirical results and robustness checks. The “Mechanism analysis” section examines the mechanisms of impact. The “Further analysis” section presents additional analyses. Finally, the “Conclusion” section concludes this study.

### Literature review and research hypothesis

The vigorous development of intelligent manufacturing has driven the emergence of advanced business models and industries, continuously injecting new vitality into the manufacturing sector [26]. Developed countries such as the United States, Germany, France, Japan, the United Kingdom, and Italy have listed intelligent manufacturing as a national strategy for industrial upgrades and technological innovation [3]. In recent years, many scholars have focused on the impact of digital and intelligent technologies on manufacturing enterprises. Ding et al. [27] used data from Chinese A-share manufacturing listed companies from 2009 to 2021 and found that digital transformation is conducive to improving firms’ TFP, and that the improvement in ESG performance owing to digital transformation also contributes to the enhancement of TFP. Yang et al. [28] found that intelligent manufacturing has a significant positive effect on firms’ financial and innovation performance. Ying et al. [29] explored the mechanisms underlying the impact of intelligent manufacturing on firm innovation and found that intelligent manufacturing promotes organizational learning by improving the learning environment, learner quality, and organizational learning investment, thereby enhancing firms’ innovation input and output.

However, some scholars argue that the “Solow Paradox” persists in the digital economy era [30] and intelligent manufacturing policies might not generate positive economic effects. Wen and Zhao [21] discovered that although an intelligent manufacturing strategy increases firms’ R&D investment, it does not have a significant impact on innovation output or TFP in the short term. Liu et al. [22] examined the effect of intelligent manufacturing policies on the asset prices of Chinese firms and found that, post-implementation, the profitability of Chinese firms decreased notably. They concluded that the policies provoked only short-term market reactions without promoting the long-term development of the targeted industries. Li and Branstetter [31] assessed the effectiveness of intelligent manufacturing policies and discovered that these policies resulted in more innovation subsidies for the targeted listed companies but found almost no statistical evidence indicating improvements in productivity or innovation capabilities. The implementation of intelligent manufacturing policies may also pose potential risks or challenges. Intelligent manufacturing may produce a substitution effect on labor, and excessive automation could lead to resource misallocation and labor underutilization, thus hindering productivity improvements [32]. The mismatch between technology and skilled labor may slow the adaptation of labor demand, diminishing the productivity gains from intelligent manufacturing. Furthermore, as it is difficult to accurately identify new industries with potential comparative advantages and technological progress beforehand, and the government’s response to the market is often sluggish, intelligent manufacturing policies may be misused to protect declining industries [33].

Therefore, the economic impact of intelligent manufacturing policies on firms remains unclear. Additionally, there is a scarcity of literature directly examining the relationship between intelligent manufacturing and TFP, with most studies focusing on the effects of intelligent manufacturing on technological innovation. In reality, intelligent manufacturing can improve firms’ ESG performance [34], and higher ESG ratings can enhance firms’ TFP [19, 20]. Therefore, this study proposes that intelligent manufacturing policies influence firms’ TFP by influencing their ESG practices.

First, intelligent manufacturing, characterized by technological advancement, helps firms enhance their green technology innovation capabilities, thereby positively impacting their ESG performance. On one hand, firms leveraging advanced technologies such as big data and artificial intelligence in intelligent manufacturing can accurately identify environmental pollution issues faced by enterprises, society, and the nation, and gain deep insights into customers’ demand for green products. With this precise information, firms can formulate more accurate green innovation plans [35], driving a shift from experience-driven to data-driven green technology innovation. On the other hand, technologies like artificial intelligence and cloud computing, which underpin intelligent manufacturing, not only promote the flow of information and knowledge within firms but also help firms transcend “time and space limitations” to form knowledge innovation networks with external entities [36], further facilitating green innovation. Thus, by enhancing green innovation capabilities, intelligent manufacturing aids firms in effectively fulfilling low-carbon environmental responsibilities, achieving comprehensive value enhancement in the economic, social, and environmental dimensions, and ultimately improving their ESG performance.

Moreover, intelligent manufacturing policies can increase the attention of the capital market, thus enhancing the market pressure faced by firms and increasing their willingness to undertake ESG practices. The rollout and implementation of intelligent manufacturing policies highlighted the significance and future trajectory of this sector, providing clear investment guidance to capital markets. Capital markets, being forward-looking and directive, fully consider policy influences when evaluating firm value. Firms backed by such policies generally have greater profitability and growth potential, thereby attracting more market attention. Increased analyst attention makes it more difficult for management to conceal negative information, effectively reducing earnings management motives and limiting opportunistic behavior [37]. Specifically, if analysts find that a listed company engages in “greenwashing,” fake social responsibility actions, or has been fined for environmental issues, its valuation in the capital market will decrease. This feedback mechanism can constrain short-term behavior to some extent, encouraging firms to prioritize sustainable development, and boosting their willingness to engage in ESG practices.

Finally, intelligent manufacturing, characterized by improved resource allocation efficiency, helps reduce internal control costs, thereby enhancing firms’ ESG performance. On one hand, internal control costs are a significant representation of agency problems. The greater the conflict of interest between shareholders and management, the higher the agency costs and risks. The implementation of intelligent manufacturing policies guides firms in undertaking intelligent transformations, helping them achieve transparency and efficiency in internal information flows, reducing the motivation for opportunistic behaviors of internal managers, and consequently lowering agency costs and risks. However, intelligent manufacturing significantly reduces costs and increases efficiency [38]. Intelligent manufacturing enables refined, flexible, and intelligent process management, effectively addressing the high management expenses typical of traditional firms, and improving corporate governance efficiency. Additionally, a reduction in management costs allows firms to allocate more funds to ESG-related investments, thereby directly promoting improvements in ESG performance.

H1a: The implementation of intelligent manufacturing policies can enhance firms’ ESG performance by strengthening green technology innovation capabilities.
H1b: The implementation of intelligent manufacturing policies can enhance firms’ ESG performance by increasing capital market attention.
H1c: The implementation of intelligent manufacturing policies can enhance firms’ ESG performance by reducing internal control costs.

Ample literature has empirically investigated the relationship between corporate ESG and TFP, finding that higher corporate ESG ratings contribute to improved TFP [19, 20, 39, 40]. According to signaling and reputation theory, ESG information disclosure has a signaling effect, sending positive signals to the outside world that the company prioritizes for sustainable development. This helps enhance a company’s social reputation and image [41], attract outstanding talent from various sectors [42], and secure more resource support. Companies with high ESG ratings are often more likely to obtain external financing from financial institutions and receive government subsidies or policy support [43, 44]. ESG ratings can also assist management in more comprehensively assessing the situation faced by a company, reducing overinvestment driven by personal interests, and allocating limited resources to more efficient projects, thereby improving investment efficiency [27]. Thus, the implementation of intelligent manufacturing policies can improve listed companies’ ESG performance, thereby optimizing resource allocation efficiency and enhancing TFP. Therefore, we propose the following hypothesis:

H2: The implementation of intelligent manufacturing policies can improve firms’ ESG performance, thereby enhancing firms’ TFP.

### Research design

#### Data source and processing

This study selects A-share listed manufacturing companies in China from 2010 to 2023 to explore the impact of intelligent manufacturing policies on firms’ TFP. Although the state promotes intelligent manufacturing policies, only a few listed companies disclose subsidy information directly related to intelligent manufacturing development strategy [31]. This indicates that corporate-level intelligent manufacturing-related information disclosure is limited, making it difficult to accurately identify the actual situation of intelligent manufacturing development strategies. Thus, this study matches the top 10 key industry sectors targeted by the intelligent manufacturing policy with the industry classification of the China Securities Regulatory Commission to identify the industries and corresponding companies implementing intelligent manufacturing. The ten key areas covered by “Made in China 2025” include next-generation information technology, high-end CNC machine tools and robots, aerospace equipment, marine engineering equipment and high-tech ships, advanced rail transportation equipment, energy-saving and new energy vehicles, power equipment, new materials, biomedicine and high-performance medical devices, and agricultural machinery equipment. The research sample is processed as follows: (1) ST and *ST companies are excluded; (2) the sample companies are restricted to the manufacturing industry; (3) samples with missing data on core variables are excluded; and (4) continuous variables are winsorized at the 1% and 99% levels. Employee skill data are obtained from the Wind database, whereas other financial data are sourced from the CSMAR and CNRDS databases.

### Variable definitions

#### Total factor productivity (TFP)

This study employs the LP method [45] to estimate firms’ TFP. Specifically, output is measured using operating revenue, labor input is measured using the number of employees, capital stock is measured using the net value of fixed assets, and intermediate input is measured using cash paid for goods and services. Additionally, the OP [46] and Wooldridge methods [47] are used to recalculate firm TFP to verify the robustness of the empirical results.

#### ESG performance *(ESG)*

This study uses the Huazheng ESG rating as a proxy for corporate ESG performance. The Huazheng ESG rating assigns ratings from “AAA” to “C” in nine levels, covering all A-share listed companies and widely recognized by industry and academia. This study quantifies the ESG levels of listed companies, assigning values from 1 to 9, with 1 being the lowest “C” level and 9 being the highest “AAA” level. The higher the rating score, the better the corporate ESG performance.

#### Intelligent manufacturing policy (*Treat* × *Post*)

The core explanatory variable of this study is *Treat*_*j*_×*Post*_*t*_, which is a dummy variable for intelligent manufacturing policy. *Treat*_*j*_ is the group dummy variable that takes the value of 1 if the company’s industry belongs to the top 10 key manufacturing sectors and 0 otherwise. *Post*_*t*_ is the time dummy variable, which takes the value of 1 for the years 2015 and beyond, marking the release year of “Made in China 2025,” and 0 for the years before 2015.

#### Control variables

Based on relevant literature and data availability, this study controls for other firm-level characteristics that may affect corporate TFP, specifically including: firm age (*Age*), measured by the natural logarithm of the number of years since establishment; historical performance (*LROA*), measured by the ratio of net profit to total assets in the previous period; leverage ratio (*Lev*), measured by the ratio of total liabilities to total assets; capital intensity (*Cap*), measured by the natural logarithm of the ratio of net fixed assets to the number of employees; cash flow level (*Cash*), measured by the ratio of net cash flow from operating activities to total assets; management shareholding ratio (*MSR*), measured by the proportion of shares held by management; ownership concentration (*Share*), measured by the proportion of shares held by the largest shareholder; growth (*Growth*), measured by the growth rate of operating income; financing constraints (*FC*), measured by the SA index constructed by Hadlock and Pierce [48]; firm value (*Tbq*), measured by Tobin’s Q; asset turnover rate (*Tur*), measured by the ratio of operating income to total assets. Table 1 presents the definitions of the main variables.

**Table 1:** Variable definitions.

Variables	Description
*TFP_LP*	Firm TFP calculated by using the LP method
*ESG*	The ESG rating index of Huazheng
*Treat*	Enterprises in key areas of intelligent manufacturing are assigned a value of 1, otherwise 0
*Post*	Year greater than or equal to 2015 is assigned a value of 1, otherwise 0
*Age*	Natural logarithm of the years since the establishment of the enterprise
*LROA*	Ratio of net profit to total assets in the previous period
*Lev*	Ratio of total liabilities to total assets
*Cap*	Natural logarithm of the ratio of net fixed assets to total number of employees
*Cash*	Ratio of net cash flow from operating activities to total assets
*MSR*	Management shareholding ratio
*Share*	Shareholding ratio of the largest shareholder
*Growth*	Growth rate of operating revenue
*FC*	SA index constructed by Hadlock and Pierce [48]
*Tbq*	Tobin’s Q ratio
*Tur*	Ratio of operating revenue to total assets

#### Model specification

To examine the impact of intelligent manufacturing policies on firms’ TFP, this study constructs the following difference-in-differences (DID) model:

$${TFP}_{it}=\beta_{0}+\beta_{1}{Treat}_{j}\times {Post}_{t}+\beta_{2}{Controls}_{it}+\tau_{i}+\mu_{t}+\varepsilon_{it}$$ (1)

where the dependent variable *TFP*_*it*_ represents the TFP of firm *i* in year *t*. The explanatory variable *Treat*_*j*_ is a dummy variable that takes the value of 1 if the industry to which the firm belongs is one of the 10 key manufacturing sectors; otherwise, it takes the value of 0; *Post*_*t*_ represents the time of implementation of the intelligent manufacturing policy, defined as 1 for 2015 and thereafter, and 0 otherwise. This study focuses on the sign of the coefficient of the interaction term *Treat*_*j*_×*Post*_*t*_. If *β*_1_ is significantly positive, it indicates that the implementation of the intelligent manufacturing policy can promote the improvement of firms’ TFP. *Controls*_*it*_ represents a set of control variables. *τ*_*i*_ represents firm fixed effects; *μ*_*t*_ represents year fixed effects; *ε*_*it*_ represents random disturbance terms.

We then study the relationship between intelligent manufacturing policies and firms’ ESG performance and examine whether intelligent manufacturing policies affect firms’ TFP by influencing their ESG performance. Accordingly, we construct the following models:

$${ESG}_{it}=\beta_{0}+\beta_{1}{Treat}_{j}\times {Post}_{t}+\beta_{2}{Controls}_{it}+\tau_{i}+\mu_{t}+\varepsilon_{it}$$ (2)

$${TFP}_{it}=\beta_{0}+\beta_{1}{Treat}_{j}\times {Post}_{t}+\beta_{2}{ESG}_{it}+\beta_{3}{Controls}_{it}+\tau_{i}+\mu_{t}+\varepsilon_{it}$$ (3)

#### Descriptive statistics

The sample comprises 18 682 observations from 2736 firms. Table 2 presents the descriptive statistics for the main variables. The mean of TFP (*TFP_LP*) calculated using the LP method for listed firms is 15.4597, and the median is 16.1779, indicating no significant skewness in the data; the standard deviation is 2.6447, suggesting considerable variation in TFP among different firms. The mean of the dummy variable *Treat* × *Post* is 0.5723, indicating that 57.23% of the observations come from the period after the implementation of the intelligent manufacturing policy. Data distribution was reasonable and met the research requirements. For the control variables, characteristics such as *Age*, *Lev*, *Cap*, *Cash*, and *Growth* are realistic and generally consistent with previous studies.

**Table 2:** Descriptive statistics.

Variable	N	Mean	S.D.	Min	Median	Max
*TFP_LP*	18682	15.4597	2.6447	8.4889	16.1779	20.5907
*ESG*	18682	4.1569	0.8540	1.0000	4.0000	8.0000
*Treat*×*Post*	18682	0.5723	0.4948	0.0000	1.0000	1.0000
*Age*	18682	2.8445	0.3431	1.6094	2.8904	3.4965
*LROA*	18682	0.0521	0.0540	-0.1558	0.0483	0.2127
*Lev*	18682	0.3792	0.1813	0.0491	0.3723	0.7813
*Cap*	18682	12.6356	0.8373	10.2084	12.6300	14.7335
*Cash*	18682	0.0541	0.0642	-0.1287	0.0511	0.2466
*MSR*	18682	15.6725	20.3522	0.0000	3.2426	69.9144
*Share*	18682	33.5686	14.1194	8.8621	31.6045	73.3158
*Growth*	18682	0.1601	0.3005	-0.4430	0.1159	1.5762
*FC*	18682	-3.8215	0.2376	-4.4341	-3.8225	-3.2137
*Tbq*	18682	2.2359	1.3179	0.9148	1.8102	8.2373
*Tur*	18682	0.6319	0.3410	0.1299	0.5620	2.1124

### Empirical results

#### Baseline regression analysis results

Table 3 reports the empirical results of the impact of intelligent manufacturing policies on firms’ TFP. Column (1) presents the regression analysis results without any control variables, where the coefficient of the core explanatory variable *Treat* × *Post* is significantly positive at the 1% level, indicating that firms affected by intelligent manufacturing policies have higher TFP. This study further includes control variables in the regression analysis, as shown in Column (2), where the coefficient of *Treat* × *Post* is 0.0913 and is significant at the 1% level. Moreover, using Column (2) with all control variables as an example, the paper illustrates the economic significance of the estimated results. The coefficient suggests that firms affected by the intelligent manufacturing policy have, on average, a 9.13% increase in TFP compared to unaffected firms. To further clarify the policy’s impact on TFP, we calculated the standard deviation change in TFP (*Treat* × *Post* coefficient divided by the *TFP_LP* standard deviation) and found that the policy led to an approximate increase of 0.0345 standard deviations in TFP, roughly corresponding to 3.45% of the total TFP variation during the period. In summary, the intelligent manufacturing policy’s impact on firms’ TFP is not only statistically significant but also economically meaningful.

**Table 3:** The impact of intelligent manufacturing policies on firm TFP.

	(1)	(2)
	*TFP_LP*	*TFP_LP*
*Treat*×*Post*	0.1138***	0.0913***
	(0.0283)	(0.0223)
*Age*		0.4157***
		(0.0859)
*LROA*		1.8082***
		(0.0851)
*Lev*		0.7668***
		(0.0544)
*Cap*		-0.0147
		(0.0145)
*Cash*		0.2633***
		(0.0530)
*MSR*		-0.0008**
		(0.0004)
*Share*		-0.0030**
		(0.0012)
*growth*		0.2621***
		(0.0120)
*FC*		0.2540*
		(0.1482)
*tbq*		-0.0218***
		(0.0044)
*tur*		0.9229***
		(0.0385)
Constant	15.0272***	14.1488***
	(0.0165)	(0.5459)
Firm FE	Yes	Yes
Year FE	Yes	Yes
N	18682	18682
R^2^	0.3413	0.6316

Note: The symbols *, **, and *** represent significance levels at 10%, 5%, and 1%, respectively. Standard errors, clustered at the firm level, are denoted in parentheses.

#### Intelligent manufacturing policy, ESG performance and TFP

To test whether intelligent manufacturing policies affect firms’ TFP by influencing their ESG performance, we conduct regression analyses based on Models (2) and (3). The results are presented in Table 4. Column (1) shows the impact of intelligent manufacturing policies on firms’ ESG performance. The coefficient of *Treat* × *Post* is significantly positive at the 1% level, suggesting that implementing intelligent manufacturing policies significantly improves firms’ ESG performance. In Column (2), the regression analysis includes intelligent manufacturing policy indicators and firms’ ESG indicators as explanatory variables. The coefficients of *Treat* × *Post* and *ESG* are significantly positive at the 1% level, suggesting that intelligent manufacturing policies enhance firms’ ESG performance, thereby improving their TFP.

**Table 4:** Intelligent manufacturing policy, ESG performance and TFP.

	(1)	(2)
	*ESG*	*TFP_LP*
*Treat*×*Post*	0.1186***	0.0874***
	(0.0394)	(0.0221)
*ESG*		0.0325***
		(0.0042)
*Age*	0.0355	0.4145***
	(0.1474)	(0.0854)
*LROA*	1.8188***	1.7491***
	(0.1881)	(0.0827)
*Lev*	-0.2308**	0.7742***
	(0.0905)	(0.0540)
*Cap*	-0.0473**	-0.0131
	(0.0205)	(0.0145)
*Cash*	-0.0202	0.2639***
	(0.1207)	(0.0527)
*MSR*	0.0029***	-0.0009**
	(0.0008)	(0.0004)
*Share*	-0.0017	-0.0029**
	(0.0016)	(0.0012)
*growth*	0.0030	0.2620***
	(0.0228)	(0.0119)
*FC*	0.7037***	0.2312
	(0.2201)	(0.1462)
*tbq*	-0.0227***	-0.0211***
	(0.0080)	(0.0043)
*tur*	-0.0840	0.9256***
	(0.0549)	(0.0382)
Constant	7.1834***	13.9156***
	(0.8693)	(0.5380)
Firm FE	Yes	Yes
Year FE	Yes	Yes
N	18682	18682
R^2^	0.0368	0.6349

Note: The symbols *, **, and *** represent significance levels at 10%, 5%, and 1%, respectively. Standard errors, clustered at the firm level, are denoted in parentheses.

#### Endogenous treatment

In general, firm behavior does not affect the implementation of intelligent manufacturing policies. Logically, there is no significant reverse causality issue. However, since the effect of intelligent manufacturing policies on firms’ ESG performance and TFP may be influenced by other unobservable factors, the current results could be biased owing to endogeneity issues. Therefore, this study uses the instrumental variable (IV) method to alleviate potential endogeneity problems. Specifically, this study selects the number of employees in the telecommunications and other information transmission service industries in the region where the firm was located in 2003 as the instrumental variable for intelligent manufacturing [49]. On one hand, the historical development level of the telecommunications and other information transmission services industries in a region is unlikely to directly impact firms’ ESG performance and TFP, satisfying the exogeneity requirement of the IV. On the other hand, the execution of intelligent manufacturing policies and intelligent transformation of firms depends on the support of regional network infrastructure. The historical development level of the telecommunications and other information transmission service industries directly affects the subsequent development and spread of local network technology, meeting the relevance requirements of the IV. Notably, the original data of the chosen IV are in a cross-sectional form and cannot be used directly in the panel data regression analysis. Therefore, this study uses the product of the ratio of industry employment to national manufacturing employment and the number of employees in the telecommunications and other information transmission service industries in each region in 2003 as the instrumental variable (*IV1*) for intelligent manufacturing.

Table 5 presents the estimation results of the two-stage least squares (2SLS) method. Column (1) shows the first-stage regression analysis results. The *IV1* coefficient is significantly positive at the 1% level, indicating that the chosen instrumental variable is highly positively correlated with the intelligent manufacturing policy variable. Column (2) displays the second-stage regression analysis results, where the coefficient of *Treat* × *Post* is significantly positive at the 1% level, suggesting that the positive effect of intelligent manufacturing policies on firms’ TFP remains after considering the endogeneity issues. The results of the instrumental variable relevance tests show that the Kleibergen-Paap rk LM statistic is 209.561 (P-value = 0.000), rejecting the null hypothesis of under-identification, and the Kleibergen-Paap rk Wald F statistic is 292.195, which is greater than the critical value at the 10% level (16.38), signifying the absence of weak instrumental variable problems.

**Table 5:** Instrumental variable analysis.

	(1)	(2)	(3)	(4)	(5)	(6)
	*Treat×Post*	*ESG*	*TFP_LP*	*Treat×Post*	*ESG*	*TFP_LP*
*IV1*	0.7311***					
	(0.0428)					
*IV2*				0.0542***		
				(0.0036)		
*Treat*×*Post*		1.3787***	0.7177***		1.3073***	0.7386***
		(0.1800)	(0.1192)		(0.1983)	(0.1402)
*Controls*	Yes	Yes	Yes	Yes	Yes	Yes
Firm FE	Yes	Yes	Yes	Yes	Yes	Yes
Year FE	Yes	Yes	Yes	Yes	Yes	Yes
N	18305	18305	18305	15161	15161	15161
K-P rk LM	209.561			180.971		
K-P Wald rk F	292.195			228.933		

Note: The symbols *, **, and *** represent significance levels at 10%, 5%, and 1%, respectively. Standard errors, clustered at the firm level, are denoted in parentheses.

Furthermore, to ensure the robustness of the IV estimation results, this study selects the interaction term between the number of post offices per million people in 1984 cities and the ratio of industry employment to national manufacturing employment as the second instrumental variable (*IV2*) [50]. The distribution of post offices influenced the early use of internet technologies and habits, which later impacted the development and dissemination of intelligent manufacturing technologies. From this perspective, the choice of post office numbers as an IV meets the relevance criterion. Meanwhile, the historical number of post offices has no direct connection with the current ESG performance or TFP of manufacturing firms, thereby satisfying the exogeneity criterion of the IV. As shown in Columns (4) to (6) of Table 5, the estimation results indicate that intelligent manufacturing policies still improve firms’ ESG performance and TFP, with the under-identification test and weak instrumental variable test significantly rejecting the null hypothesis.

### Robustness test

To further validate the positive impact of intelligent manufacturing policies on firms’ TFP, this study conducts robustness analyses using parallel trend tests, placebo tests, and alternative measures of key variables, among others. The specific test results are as follows:

### Parallel trend test

**Fig 1:**
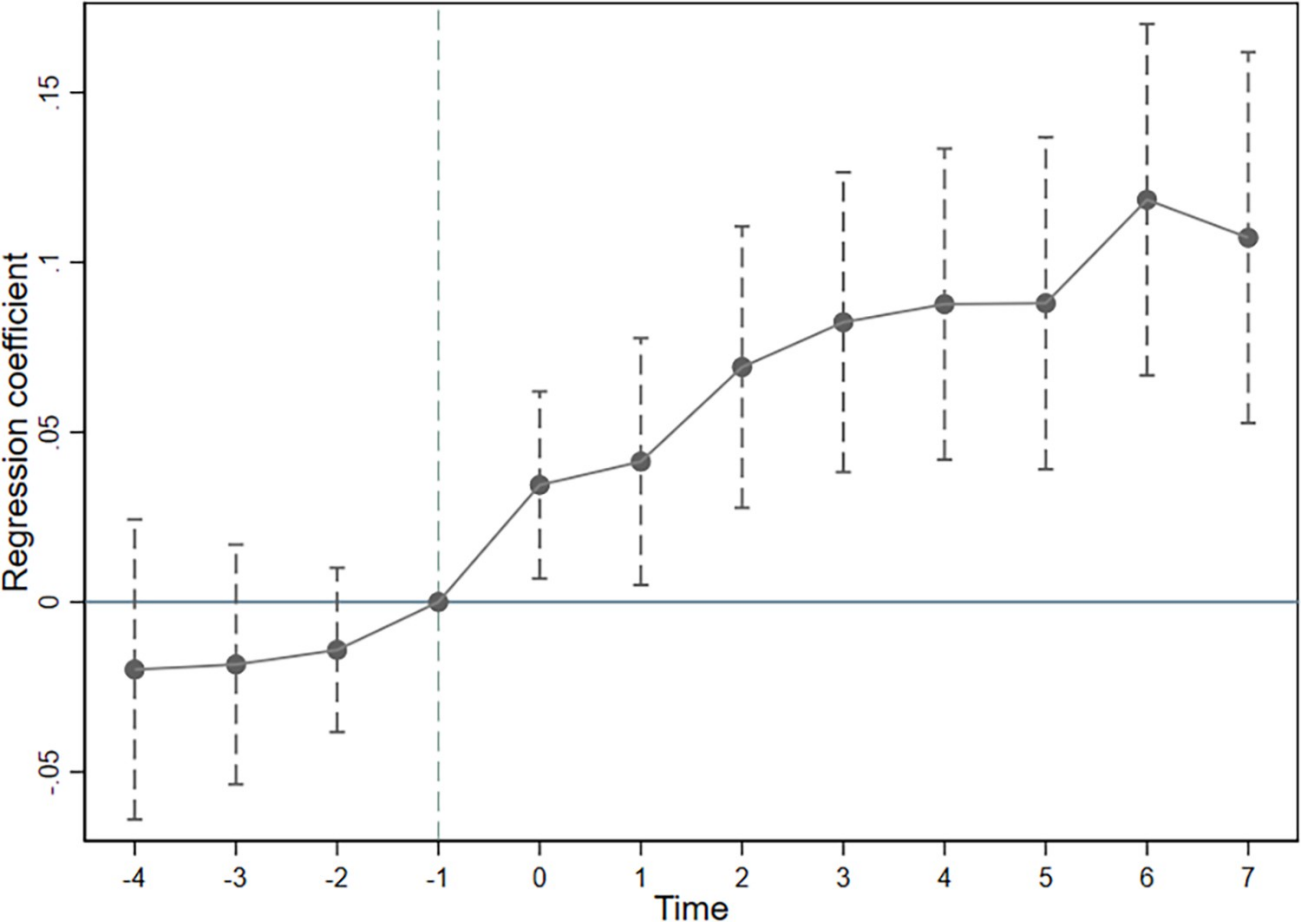
Parallel trend test.

Parallel trend assumption is a prerequisite for the DID model to correctly identify causal relationships. Moreover, the estimation results in Table 3 reflect only the overall effect of intelligent manufacturing policies on firm TFP. To further test the parallel trend assumption and examine the annual effects of firms’ TFP before and after the implementation of intelligent manufacturing policies, we construct the following regression equation:

$${TFP}_{it}=\alpha +\sum_{k}\beta_{k}\times ({Treat}_{j}\times Yr_{k})+\gamma Controls+\tau_{i}+\mu_{t}+\varepsilon_{it}$$ (4)

where *Yr*_*k*_ is a dummy variable that takes the value of 1 if the observation time is the kth year before the implementation of the intelligent manufacturing policy, and 0 otherwise. The other variable definitions are consistent with those in Model (1). To avoid multicollinearity, the year before the implementation of the intelligent manufacturing policy was used as the baseline. Fig 1 shows the estimation results of Model (4). This shows that before the implementation of the intelligent manufacturing policy, the estimated coefficients are not significantly different from 0, indicating no significant difference in the trend of firms’ TFP between the treatment and control groups before the policy, which is consistent with the parallel trend assumption. After the implementation of the intelligent manufacturing policy, the estimated coefficients were significantly positive, indicating that the policy significantly improved firms’ TFP, which is consistent with previous research.

### Placebo tests

**Fig 2:**
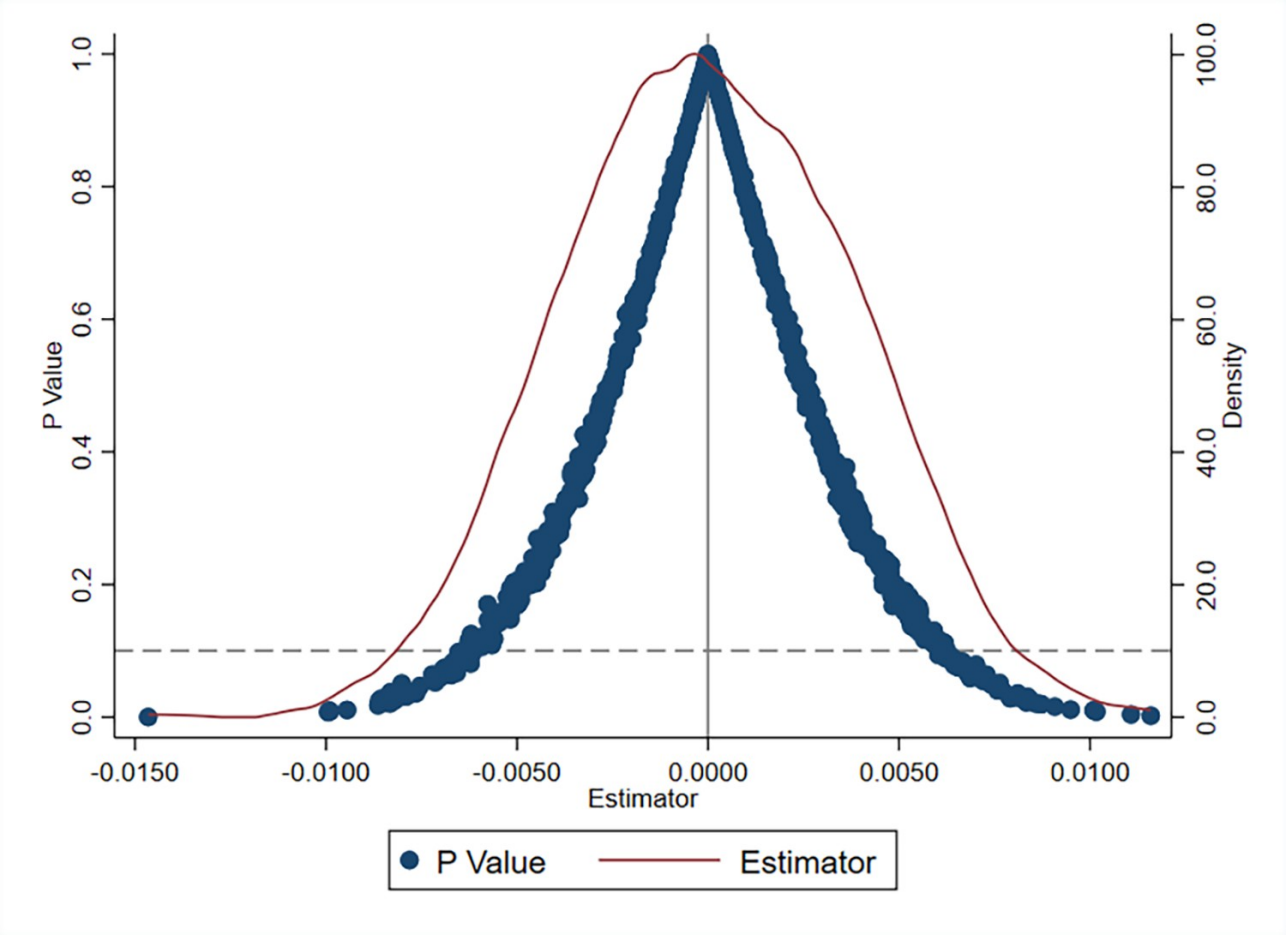
Placebo tests.

To verify that the impact of intelligent manufacturing policies on firms’ TFP was not owing to other random factors, we employed a placebo test to identify the fortuitousness of the intelligent manufacturing policy effect. Specifically, by randomly setting the policy implementation time, the policy dummy variable is restructured and the restructured false policy dummy variable *Treat*_*j*_×*Post*_*j*_ is substituted into the benchmark regression Model (1) for regression. This process was repeated 1,000 times and a distribution plot of the regression coefficients of *Treat*_*j*_×*Post*_*j*_ from the 1,000 regression analyses is generated. The results in Fig 2 show that the estimated coefficients of the false policy variable approximately follow a normal distribution with a mean of 0, and most of the estimated p-values are greater than 0.1. Combined with the regression coefficient of 0.0913 for the impact of intelligent manufacturing policies on firms’ TFP in Column (2) of Table 3, the random simulation of policy shocks does not significantly impact firms’ TFP. This analysis demonstrates that the positive effect of intelligent manufacturing policies on firms’ TFP is not owing to other random factors.

### Alternative measures of key variables

(1) Replacing the dependent variable: Considering that using only the LP method to calculate firms’ TFP may introduce errors, this study recalculates firms’ TFP using the OP [46] and Wooldridge methods [47] and conducts regression analyses based on Model (1). The regression analysis results in Columns (1) and (2) of Table 6 show that the coefficients of *Treat* × *Post* remain significantly positive at the 1% level. This indicates that even with different methods of calculating firms’ TFP, the conclusion that intelligent manufacturing policies enhance firms’ TFP remains robust. (2) Replacing the intelligent manufacturing policy metric: To rule out the interference of the Intelligent Manufacturing policy measurement index on the research conclusions, this study uses the “Intelligent Manufacturing 2025” concept stock firms to measure the impact of intelligent manufacturing policies [22]. The regression analysis results are shown in Column (3) of Table 6, where the coefficient of *Treat*_*1*_ × *Post* is significantly positive, confirming the robustness of the previous conclusions.

**Table 6:** Robustness test: Alternative measures of key variables and PSM-DID test.

	(1)	(2)	(3)	(4)
	*TFP_OP*	*TFP_WRDG*	*TFP_LP*	*TFP_LP*
*Treat*×*Post*	0.1512***	0.0886***		0.0915***
	(0.0206)	(0.0219)		(0.0223)
*Treat*_*1*_×*Post*			0.0781*	
			(0.0474)	
Constant	11.8239***	13.8783***	14.0756***	14.1455***
	(0.4855)	(0.5380)	(0.5513)	(0.5459)
*Controls*	Yes	Yes	Yes	Yes
Firm FE	Yes	Yes	Yes	Yes
Year FE	Yes	Yes	Yes	Yes
N	17476	18682	18682	18679
R^2^	0.6287	0.6319	0.6302	0.6316

Note: The symbols *, **, and *** represent significance levels at 10%, 5%, and 1%, respectively. Standard errors, clustered at the firm level, are denoted in parentheses.

### PSM-DID test

Considering that significant differences in observable firm characteristics between the treatment and control groups before the implementation of intelligent manufacturing policies could lead to sample selection bias in the estimated results, this study further employs the propensity score matching (PSM) method. This approach identifies samples from the control group with characteristics similar to those of the treatment group to mitigate sample selection bias caused by firm heterogeneity. Specifically, the k-nearest neighbor matching method was used with a matching ratio of 1:1, with firm age, historical performance, leverage, capital intensity, cash flow level, managerial ownership, ownership concentration, growth, financing constraints, firm value, and asset turnover ratio as covariates for matching. Based on samples matched using the PSM method, this study retests the impact of intelligent manufacturing policies on firms’ TFP using the DID model. The regression analysis results are presented in Column (4) of Table 6. The results show that the coefficient of *Treat* × *Post* remains positive and significant at the 1% level, indicating that implementing intelligent manufacturing policies enhances firms’ TFP.

### Controlling for the impact of potential omitted variables

To address potential omitted variable bias, this study further incorporates control variables into the baseline regression, including government subsidies (*pubsub*) and international trade (*fdi*), which may influence firms’ TFP. Specifically, *pubsub* represents the total amount of various types of government subsidies received by listed companies, while *fdi* reflects the actual amount of foreign direct investment utilized in each city during the year. The test results, presented in Column (1) of Table 7, show that the coefficient of *Treat* × *Post* remains significantly positive at the 1% level, affirming the robustness of the study’s conclusions.

**Table 7:** Robustness test: Excluding the interference of other factors.

	(1)	(2)	(3)
	*TFP_LP*	*TFP_LP*	*TFP_LP*
*Treat*×*Post*	0.0769***	0.0924***	0.0954***
	(0.0221)	(0.0222)	(0.0244)
*broadband*			-0.0008
			(0.0210)
Constant	12.9604***	14.6145***	14.3394***
	(0.7419)	(0.5745)	(0.5725)
*Controls*	Yes	Yes	Yes
Firm FE	Yes	Yes	Yes
Year FE	Yes	Yes	Yes
N	9941	16231	16359
R^2^	0.6195	0.6381	0.6292

Note: The symbols *, **, and *** represent significance levels at 10%, 5%, and 1%, respectively. Standard errors, clustered at the firm level, are denoted in parentheses.

### Excluding the interference of other policies

To ensure the robustness of the study’s findings, this study also attempts to rule out the impact of other significant policies implemented during the sample period. In October 2010, the State Council released the “Decision on Accelerating the Cultivation and Development of Strategic Emerging Industries,” and in 2012, the National Bureau of Statistics issued the “Strategic Emerging Industries Classification (2012),” which clarified the definition of these industries. Later, the National Development and Reform Commission introduced several related policies to foster the growth of strategic emerging industries. These policies may have affected the results of this study. As a result, firms influenced by strategic emerging industry policies were excluded for robustness testing. The results, as presented in Column (2) of Table 7, show that even after excluding the influence of strategic emerging industry policies, the positive effect of intelligent manufacturing policies on firms’ TFP remains robust.

In August 2013, the State Council issued the “Broadband China” Strategy and Implementation Plan, aimed at promoting the construction of China’s broadband network infrastructure. The “Broadband China” policy could promote firms’ TFP growth by improving the level of network infrastructure [51]. To eliminate the interference of the “Broadband China” policy, this study adds a "Broadband China" policy variable (broadband) to the baseline regression. The results, shown in Column (3) of Table 7, indicate that after controlling for the influence of the "Broadband China" policy, intelligent manufacturing policies still promote firms’ TFP.

### Mechanism analysis

Based on the previous conclusions, the implementation of intelligent manufacturing policies is conducive to enhancing firms’ ESG performance, thereby improving firms’ TFP. Green technology innovation not only helps firms reduce pollutant emissions and improve resource efficiency but also enables them to develop more environmentally friendly products and services, thereby directly enhancing their environmental performance. The heightened attention from capital markets can motivate firms to actively fulfill their social responsibilities and strengthen corporate governance to maintain their image and stabilize stock prices. Effective internal control can reduce operational risks, improve decision-making efficiency, and mitigate opportunistic behavior by internal managers, thus enhancing governance performance. Therefore, this study posits that intelligent manufacturing policies can influence firms’ ESG performance through three channels: green technology innovation, capital market attention, and internal control. Firms’ improved ESG performance further enhances their TFP.

### Green technology innovation

The theoretical analysis indicates that firms utilizing advanced technologies such as big data and artificial intelligence in intelligent manufacturing development can accurately grasp the environmental pollution issues faced by firms, society, and the state, as well as the green product demands of corporate clients, making green technology innovation activities more efficient and precise. Additionally, intelligent manufacturing, driven by data and data flow, strengthens information-sharing and knowledge-integration within and between firms, thereby providing strong support for green innovation. This study posits that green innovation activities driven by intelligent manufacturing policies can provide a solid foundation for technological development. Further, while promoting green environmental governance and fulfilling social responsibilities, these activities can cultivate firms’ unique green competitiveness, and enhance their ESG performance and TFP.

To verify this hypothesis, we use the number of green patent applications to measure firms’ green technology innovation [52]. To ensure that the patent application numbers are as normally distributed as possible, this study takes the natural logarithm of the number of green patents plus one, yielding the number of green invention patent applications (*Inno1*) and the number of green utility model patent applications (*Inno2*). Table 8 presents the results of the mechanism test for green technology innovation. Columns (1) and (2) show that the coefficients of *Treat* × *Post* are significantly positive at the 1% and 5% levels, respectively, indicating that intelligent manufacturing policies promote firms’ green technology innovation. The regression analysis results in Columns (3)–(6) show that when the dependent variables are firms’ ESG performance and TFP, the coefficients of *Treat* × *Post* and green technology innovation (*Inno1*, *Inno2*) are significantly positive at the 1% level. Combined with previous conclusions, this proves that intelligent manufacturing policies can enhance firms’ green innovation capabilities, thereby positively affecting their ESG performance and TFP.

**Table 8:** Potential channels of green technology innovation.

	(1)	(2)	(3)	(4)	(5)	(6)
	*Inno1*	*Inno2*	*ESG*	*TFP_LP*	*ESG*	*TFP_LP*
*Treat*×*Post*	0.1783***	0.0837**	0.1091***	0.0792***	0.1157***	0.0864***
	(0.0346)	(0.0360)	(0.0393)	(0.0219)	(0.0394)	(0.0220)
*Inno1*			0.0532***	0.0675***		
			(0.0118)	(0.0062)		
*Inno2*					0.0346***	0.0581***
					(0.0112)	(0.0058)
Constant	7.3102***	4.3343***	6.7943***	13.6551***	7.0333***	13.8970***
	(0.8638)	(0.8210)	(0.8617)	(0.5260)	(0.8632)	(0.5320)
*Controls*	Yes	Yes	Yes	Yes	Yes	Yes
Firm FE	Yes	Yes	Yes	Yes	Yes	Yes
Year FE	Yes	Yes	Yes	Yes	Yes	Yes
N	18682	18682	18682	18682	18682	18682
R^2^	0.1507	0.1438	0.0386	0.6408	0.0376	0.6386

Note: The symbols *, **, and *** represent significance levels at 10%, 5%, and 1%, respectively. Standard errors, clustered at the firm level, are denoted in parentheses.

### Capital market attention

Capital markets are forward-looking and directive. The introduction and implementation of intelligent manufacturing policies highlights the importance and development direction of this field, providing a clear investment signal to the capital market. This signal significantly increases the capital market’s attention toward intelligent manufacturing-related companies, intensifying market pressure on these firms. In the long run, this pressure can help curb short-sighted corporate behavior to some extent, encouraging firms to focus more on long-term value and sustainable development. Analysts play a crucial intermediary role as information discoverers, transmitters, and predictors of future business operations in capital markets. Their deep dives and professional interpretations of corporate information allow analysts to track essential external supervision mechanisms for firms [53].

To test the impact of the “capital market attention” mechanism, this study uses analyst attention (*Analyst*) and report attention (*Report*) to measure capital market attention. Analyst attention is the natural logarithm of the number of analysts tracking a firm plus one in that year, whereas report attention is the natural logarithm of the number of reports tracking a firm plus one in that year [54]. The test results are listed in Table 9. According to Columns (1) and (2), the coefficients of *Treat* × *Post* are significantly positive at the 1% level, indicating that firms affected by intelligent manufacturing policies receive significantly higher attention from analysts and reports than those not affected by the policies. The regression analysis results in Columns (3)–(6) show that when the dependent variables are corporate ESG performance and TFP, the coefficients of *Treat* × *Post* and capital market attention (*Analyst* and *Report*) are significantly positive at the 1% level. Combined with previous conclusions, this proves that intelligent manufacturing policies effectively improve corporate ESG performance by increasing capital market attention, thereby enhancing firms’ TFP.

**Table 9:** Potential channels of capital market attention.

	(1)	(2)	(3)	(4)	(5)	(6)
	*Analyst*	*Report*	*ESG*	*TFP_LP*	*ESG*	*TFP_LP*
*Treat*×*Post*	0.1075***	0.1523***	0.1461***	0.1240***	0.1449***	0.1211***
	(0.0414)	(0.0523)	(0.0433)	(0.0219)	(0.0431)	(0.0220)
*Analyst*			0.0659***	0.0955***		
			(0.0144)	(0.0065)		
*Report*					0.0548***	0.0758***
					(0.0111)	(0.0051)
Constant	2.3614***	2.2735**	7.9816***	14.3440***	8.0197***	14.4219***
	(0.8229)	(1.0342)	(0.9483)	(0.5313)	(0.9428)	(0.5303)
*Controls*	Yes	Yes	Yes	Yes	Yes	Yes
Firm FE	Yes	Yes	Yes	Yes	Yes	Yes
Year FE	Yes	Yes	Yes	Yes	Yes	Yes
N	13282	13311	13282	13282	13311	13311
R^2^	0.1838	0.1732	0.0457	0.6993	0.0464	0.6988

Note: The symbols *, **, and *** represent significance levels at 10%, 5%, and 1%, respectively. Standard errors, clustered at the firm level, are denoted in parentheses.

### Internal control costs

Theoretical analysis indicates that intelligent manufacturing improves resource allocation efficiency and affects a firm’s internal control costs. On one hand, the implementation of intelligent manufacturing policies guides enterprises in intelligent transformation, helping them achieve transparency and efficiency in internal information flows, thereby reducing the motivation for managerial opportunism and mitigating agency problems. On the other hand, intelligent manufacturing can achieve intelligent process management and effectively address the high management costs associated with traditional enterprises. Under the combined influence of these factors, the implementation of intelligent manufacturing helps reduce a firm’s internal control costs, further enhancing ESG performance and promoting TFP growth.

To verify the validity of the internal control cost channel, we use the management expense ratio to measure a firm’s internal control costs (*Cost*) [55]. A higher management expense ratio indicates higher internal control costs. The test results are listed in Table 10. According to Column (1), the coefficient of *Treat* × *Post* is significantly negative at the 1% level, indicating that the implementation of intelligent manufacturing policies reduces internal control costs. The regression analysis results in Columns (2) and (3) show that when the dependent variables are ESG performance and TFP, the coefficient of *Treat* × *Post* is significantly positive at the 5% and 1% levels, respectively, and the coefficient of *Cost* is significantly negative at the 1% level. Combined with the previous conclusions, this demonstrates that intelligent manufacturing policies can reduce internal control costs, thereby positively affecting ESG performance and TFP.

**Table 10:** Potential channels of internal control costs.

	(1)	(2)	(3)
	*Cost*	*ESG*	*TFP_LP*
*Treat*×*Post*	-0.0096***	0.0962**	0.0566***
	(0.0017)	(0.0391)	(0.0211)
*Cost*		-2.3334***	-3.6023***
		(0.3063)	(0.1804)
Constant	0.2261***	7.7110***	14.9634***
	(0.0379)	(0.8622)	(0.5047)
*Controls*	Yes	Yes	Yes
Firm FE	Yes	Yes	Yes
Year FE	Yes	Yes	Yes
N	18682	18682	18682
R^2^	0.4165	0.0436	0.6826

Note: The symbols *, **, and *** represent significance levels at 10%, 5%, and 1%, respectively. Standard errors, clustered at the firm level, are denoted in parentheses.

## Further analysis

### The impact of internal conditions on intelligent manufacturing policies

#### Heterogeneous impact of firm size

Enterprises are vehicles for intelligent manufacturing, and the impact of intelligent manufacturing depends on firms’ characteristics. The productivity effects of intelligent manufacturing policies differ significantly across firms of varying sizes. Specifically, large enterprises with strong capital resources, technical talent reserves, and well-established markets and R&D departments can more effectively utilize intelligent technologies to promote their own intelligent transformation, thereby continuously improving their technological innovation capabilities and production efficiency. Furthermore, given their wide-ranging operations and large workforce, large enterprises can use intelligent manufacturing to effectively optimize labor structure and achieve significant cost reductions and efficiency improvements. Hence, this study posits that large enterprises are better able to absorb and translate the benefits of intelligent manufacturing policies than small- and medium-sized enterprises.

**Fig 3:**
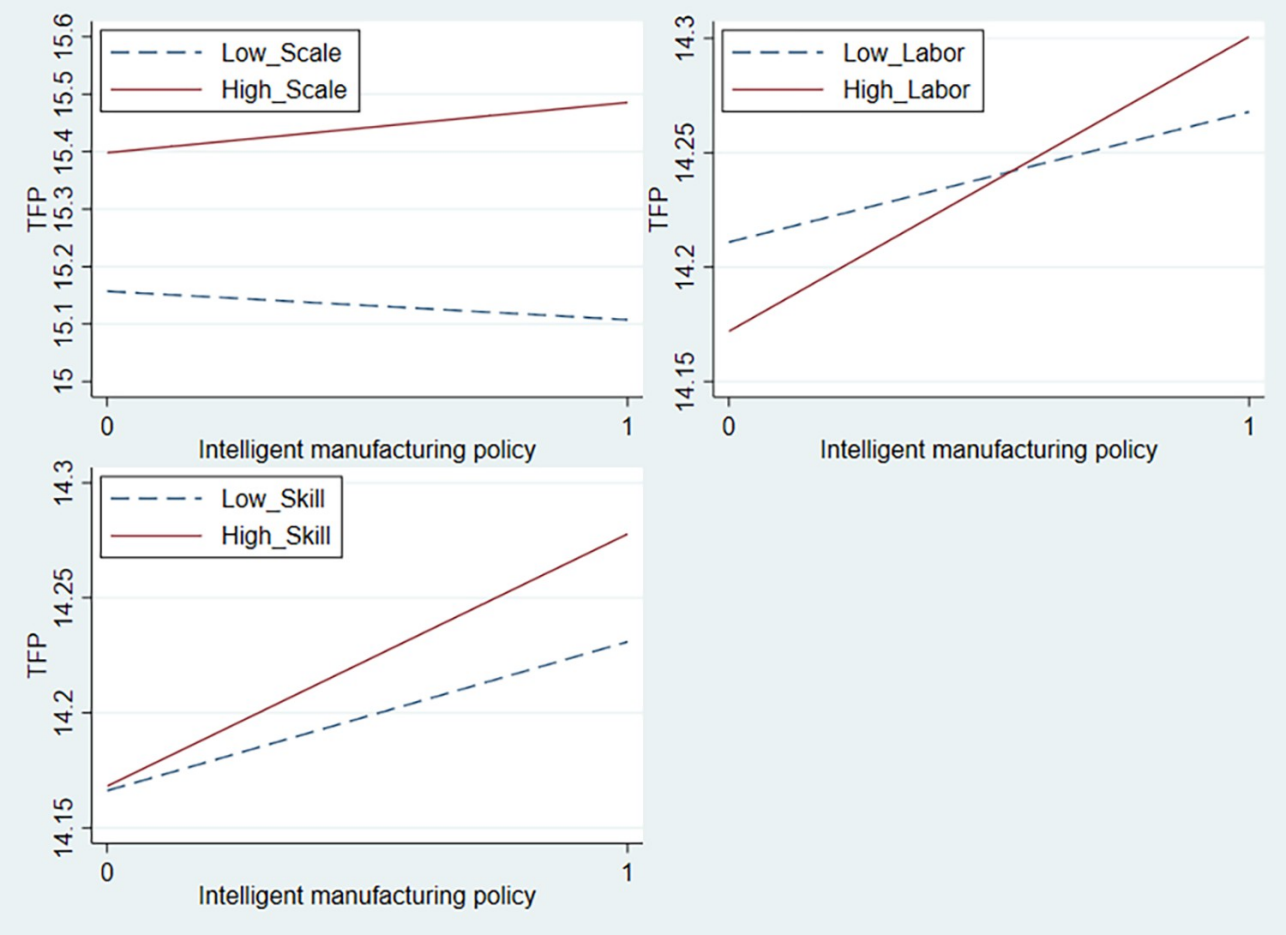
The moderating effect of internal conditions on intelligent manufacturing policies.

To define large-scale enterprises, this study follows the classification method issued by the National Bureau of Statistics, which defines enterprises with an annual operating income of 400 million yuan and above as large-scale enterprises. Therefore, we set a dummy variable for enterprise size (*Scale*), where *Scale* equals 1 for large-scale enterprises and 0 otherwise. The interaction term between intelligent manufacturing policies and enterprise size (*Treat* × *Post* × *Scale*) is included in Model (1) for examination, and the test results are shown in Column (1) of Table 11. The coefficient of *Treat* × *Post* × *Scale* is significantly positive at the 1% level, suggesting that the positive effect of intelligent manufacturing policies on TFP is more significant for large-scale enterprises, as depicted in Fig 3.

**Table 11:** Heterogeneous impact of internal conditions.

	(1)	(2)	(3)
	*TFP_LP*	*TFP_LP*	*TFP_LP*
*Treat*×*Post*×*Scale*	0.1369***		
	(0.0347)		
*Treat*×*Post*×*Labor*		0.0718***	
		(0.0194)	
*Treat*×*Post*×*Skill*			0.0449**
			(0.0174)
Constant	15.1571***	14.2109***	14.1661***
	(0.5207)	(0.5522)	(0.5445)
*Controls*	Yes	Yes	Yes
Firm FE	Yes	Yes	Yes
Year FE	Yes	Yes	Yes
N	18682	18682	18682
R^2^	0.6510	0.6329	0.6326

Note: The symbols *, **, and *** represent significance levels at 10%, 5%, and 1%, respectively. Standard errors, clustered at the firm level, are denoted in parentheses.

#### Heterogeneous impact of labor intensity

Compared to capital-intensive enterprises, labor-intensive enterprises have recently been under immense pressure from rising costs, leading them to adopt measures that increase machine operations and reduce the number of workers in an effort to cut costs and boost efficiency. Emerging digital technologies, such as industrial robots, artificial intelligence, and big data have empowered traditional production processes, with “machine substitution” displacing labor participation, thereby reducing the degree of labor involvement in traditional production and operational processes [56, 57]. Since the implementation of intelligent manufacturing policies, intelligent technologies have penetrated various industries, effectively replacing low-skilled labor, thereby significantly enhancing the production efficiency of labor-intensive enterprises. This study hypothesizes that the impact of intelligent manufacturing policies on TFP is more significant for labor-intensive enterprises.

To test this hypothesis, this study measures labor intensity as the ratio of the number of employees to fixed assets and constructs a labor intensity dummy variable (*Labor*) based on the annual median [58]. If labor intensity is greater than the median, it is assigned a value of 1; otherwise, it is assigned a value of 0. The interaction term of intelligent manufacturing policies and labor intensity (*Treat* × *Post* × *Labor*) is constructed to examine the differential impact of intelligent manufacturing policies on enterprises with different labor intensities. The results in Column (2) of Table 11 indicate that the coefficient of *Treat* × *Post* × *Labor* is significantly positive at the 1% level, suggesting that the impact of intelligent manufacturing policies on TFP is particularly significant in enterprises with higher labor intensity, consistent with the previous hypothesis. Fig 3 visually demonstrates the moderating effect of labor intensity.

#### Heterogeneous impact of employee technical level

To some extent, the effectiveness of intelligent manufacturing policies depends on the quality of the enterprise employees. Previous analyses considered enterprise employees to be homogeneous, but employees are independent individuals with different professional abilities. Generally, technical personnel have higher professional qualifications and skill levels, making them more capable of accepting and learning advanced technologies such as intelligent manufacturing, and provide the necessary skilled labor for R&D and innovation activities. Therefore, this study hypothesizes that when the proportion of technical personnel in an enterprise is higher, the effect of intelligent manufacturing policies is stronger, indicating that the impact of intelligent manufacturing policies on TFP should be more significant.

To this end, this study uses the proportion of technical personnel to measure the technical level of employees and constructs a dummy variable for employee technical level (*Skill*) according to the annual median. If the technical level of employees is greater than the median, it is assigned a value of 1; otherwise, it is assigned a value of 0. The interaction term of intelligent manufacturing policies and employee technical level (*Treat* × *Post* × *Skill*) is incorporated into Model (1) for testing, and the results are shown in Column (3) of Table 11. The coefficient of *Treat* × *Post* × *Skill* is significantly positive at the 5% level, suggesting that the effect of intelligent manufacturing policies on TFP is more significant in enterprises with more technical employees, which is consistent with the theoretical hypothesis. Fig 3 provides a more intuitive illustration of the moderating effect of employee technical level.

### The impact of the external environment on intelligent manufacturing policies

#### Heterogeneous impact of digital infrastructure

The effective implementation of intelligent manufacturing policies depends not only on internal enterprise factors but also on the external environment, particularly the level of regional digital or network infrastructure. The advancement of intelligent enterprise transformation in response to intelligent manufacturing policies requires support from the local technological environment and infrastructure, such as information access channels, to adjust production and operational activities and to innovate. Therefore, this study hypothesizes that in regions with a more-developed digital infrastructure, the positive impact of intelligent manufacturing policies on TFP is more significant.

**Fig 4:**
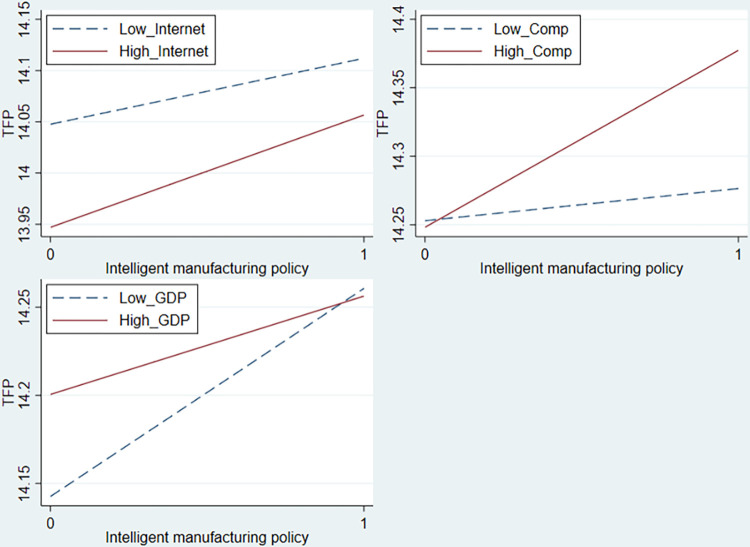
The moderating effect of the external environment on intelligent manufacturing policies.

Consequently, this study utilizes the ratio of broadband internet users to residents in a region to measure the level of digital infrastructure development [55]. Further, a dummy variable for digital infrastructure development (*Internet*) is constructed according to the annual median; if the level of digital infrastructure development is above the median, it is assigned a value of 1; otherwise, it is assigned a value of 0. The interaction term of intelligent manufacturing policies and digital infrastructure development (*Treat* × *Post* × *Internet*) is incorporated into Model (1) for testing, and the results are shown in Column (1) of Table 12. The coefficient of *Treat* × *Post* × *Internet* is significantly positive, suggesting that in regions with higher levels of digital infrastructure development, the implementation of intelligent manufacturing policies has a greater positive impact on TFP, as shown in Fig 4.

**Table 12:** Heterogeneous impact of external environment.

	(1)	(2)	(3)
	*TFP_LP*	*TFP_LP*	*TFP_LP*
*Treat*×*Post*×*Internet*	0.1927*		
	(0.1074)		
*Treat*×*Post*×*Comp*		0.6399***	
		(0.1445)	
*Treat*×*Post*×*GDP*			-0.0623**
			(0.0256)
Constant	14.1314***	14.2773***	14.1426***
	(0.5476)	(0.5646)	(0.5447)
*Controls*	Yes	Yes	Yes
Firm FE	Yes	Yes	Yes
Year FE	Yes	Yes	Yes
N	18647	18682	18682
R^2^	0.6320	0.6331	0.6325

Note: The symbols *, **, and *** represent significance levels at 10%, 5%, and 1%, respectively. Standard errors, clustered at the firm level, are denoted in parentheses.

#### Heterogeneous impact of industry competition level

Market competition compels companies to reduce information asymmetry between shareholders and management as much as possible, which helps lower internal control costs and curtail managerial opportunism. Information asymmetry and resource misallocation are more severe in less competitive industries, potentially making the impact of intelligent manufacturing policies on TFP enhancement more pronounced. However, companies in highly competitive industries face greater market pressure and are incentivized to leverage intelligent manufacturing technologies to maintain or improve their competitive advantage, indicating that these policies might also be more effective in these industries.

To elucidate the differential impact of intelligent manufacturing policies on TFP under varying levels of industry competition, this study constructs the Herfindahl-Hirschman Index (HHI) based on company revenue [59] and uses 1-HHI to measure industry competition (*Comp*), where a higher value signifies higher competition. The interaction term between intelligent manufacturing policies and industry competition (*Treat* × *Post* × *Comp*) is included in Model (1) for testing, and the results are presented in Column (2) of Table 12. The coefficient of *Treat* × *Post* × *Comp* is significantly positive at the 1% level, indicating that the positive effect of intelligent manufacturing policies on TFP is more pronounced in highly competitive industries than in industries with lower competition. This suggests that companies facing higher industry competition are more motivated to use intelligent manufacturing to enhance production efficiency, as depicted in Fig 4.

#### Heterogeneous impact of regional economic development level

Uneven regional economic development in China provides an opportunity to explore how the external environment influences the effectiveness of intelligent manufacturing policies. Owing to various factors such as history, natural resource endowment, and reform and opening-up policies, there are significant differences in economic development levels across different regions. This imbalance in economic development could lead to the varying effects of intelligent manufacturing policies on the growth, depending on the level of regional economic development. To test how regional economic development levels affect the impact of intelligent manufacturing policies, this study uses the logarithm of per capita regional GDP to measure economic development levels [60]. A dummy variable for the regional economic development level (*GDP*) is constructed based on the annual median, with a value of 1 if the economic development level is above the median and 0 otherwise. The interaction term of intelligent manufacturing policies and the economic development level (*Treat* × *Post* × *GDP*) is included in Model (1) for testing, with the results presented in Column (3) of Table 12. The coefficient of *Treat* × *Post* × *GDP* was significantly negative at the 5% level, indicating that the effect of intelligent manufacturing policies on TFP promotion is more significant in regions with lower levels of economic development. Fig 4 provides a more intuitive illustration of the moderating effect of regional economic development level. Therefore, enterprises in relatively underdeveloped regions should proactively adopt intelligent manufacturing to achieve transformation as early as possible.

## Conclusion

With the penetration of new-generation information technologies, such as artificial intelligence, big data, and cloud computing into enterprise production and manufacturing, scholars have begun to explore the impact of digital and intelligent technologies on enterprises. Against the backdrop of China’s economic growth momentum, transformation, and upgrading of the manufacturing industry, this study systematically evaluates the implementation effect of intelligent manufacturing policies. Specifically, this study uses listed manufacturing companies in China’s A-share market from 2010 to 2023 as the research sample, constructs a DID model to examine the impact of intelligent manufacturing policies on TFP, and explores the underlying mechanisms and heterogeneity factors. The implementation of intelligent manufacturing policies can significantly enhance enterprises’ ESG performance, thereby promoting TFP and driving high-quality sustainable development in manufacturing enterprises. The mechanism tests indicate that intelligent manufacturing policies improve ESG performance by strengthening green technology innovation, increasing capital market attention, and reducing internal control costs, thereby leading to TFP growth. Heterogeneity analysis reveals that the positive impact of intelligent manufacturing policies on TFP is more pronounced for large-scale enterprises, labor-intensive enterprises, enterprises with higher technical employee levels, enterprises in highly competitive industries, and enterprises located in regions with higher digital infrastructure development levels and lower economic development levels.

This study not only enriches and extends the study of the microeconomic effects of intelligent manufacturing policies but also offers the following policy implications for the intelligent development of the manufacturing industry and improvement of foundational institutions. First, the government should continuously increase its efforts to promote intelligent manufacturing in enterprises and establish a comprehensive and effective policy support system. The current findings show that the implementation of intelligent manufacturing policies effectively improves firms’ TFP. Therefore, the government should refine the policy framework, optimize the top-level design of intelligent manufacturing, and create a more favorable external environment for the robust development of intelligent manufacturing in enterprises, thereby fully realizing the growth effects of intelligent manufacturing. Second, the results reveal that the impact of intelligent manufacturing policies is affected by multiple factors, such as enterprise, industry, and regional characteristics. Consequently, enterprises should fully consider their internal and external environments when implementing intelligent manufacturing strategies and actively explore intelligent development paths tailored to their actual circumstances to leverage the enabling effects of intelligent manufacturing more effectively.

### Limitations and further research

This study has the following limitations: First, it provides evidence from Chinese manufacturing firms, so the findings may require careful consideration before being applied to countries or regions with significantly different regulatory environments, industrial structures, or stages of economic development. Second, due to the limited time span of the data, this paper may not fully capture all the effects of intelligent manufacturing policies, particularly in industries where technological and productivity changes take longer to materialize.

As for further research, several areas warrant attention. First, additional tests are needed to verify whether the results are applicable in other countries or regions. Second, given the long-term nature of ESG performance and TFP improvements, future studies should incorporate longer-term follow-up data. Third, the mechanisms through which intelligent manufacturing policies affect firms’ TFP are likely to be complex, and this study mainly focuses on the mediating role of ESG performance. Researchers could further explore other impact channels.

## Supporting information

S1 Data (XLS)

## References

[1] Koopman R, Wang Z, Wei SJ. Tracing value-added and double counting in gross exports. American Economic Review. 2014; 104(2): 459–494. doi: 10.1257/aer.104.2.459

[2] Yu C, Luo Z. What are China’s real gains within global value chains? Measuring domestic value added in China’s exports of manufactures. China Economic Review. 2018; 47: 263–273. doi: 10.1016/j.chieco.2017.08.010

[3] Zhong RY, Xu X, Klotz E, Newman ST. Intelligent manufacturing in the context of industry 4.0: a review. Engineering. 2017; 3(5): 616–630. doi: 10.1016/J.ENG.2017.05.015

[4] Tian GY, Twite G. Corporate governance, external market discipline and firm productivity. Journal of Corporate Finance. 2011; 17(3): 403–417. doi: 10.1016/j.jcorpfin.2010.12.004

[5] Raymond W, Mairesse J, Mohnen P, Palm F. Dynamic models of R & D, innovation and productivity: Panel data evidence for Dutch and French manufacturing. European Economic Review. 2015; 78: 285–306. doi: 10.1016/j.euroecorev.2015.06.002

[6] Aghion P, Cai J, Dewatripont M, Du L, Harrison A, Legros P. Industrial policy and competition. American Economic Journal: Macroeconomics. 2015; 7(4): 1–32. doi: 10.1257/mac.20120103

[7] Cassiman B, Golovko E, Martínez-Ros E. Innovation, exports and productivity. International Journal of Industrial Organization. 2010; 28(4): 372–376. doi: 10.1016/j.ijindorg.2010.03.005

[8] Chen M, Guariglia A. Internal financial constraints and firm productivity in China: Do liquidity and export behavior make a difference?. Journal of Comparative Economics. 2013; 41(4): 1123–1140. doi: 10.1016/j.jce.2013.05.003

[9] Liu S, Shen X, Jiang T, Failler P. Impacts of the financialization of manufacturing enterprises on total factor productivity: empirical examination from China’s listed companies. Green Finance. 2021; 3(1): 59–89. doi: 10.3934/gf.2021005

[10] Li K, Guo Z, Chen Q. The effect of economic policy uncertainty on enterprise total factor productivity based on financial mismatch: Evidence from China. Pacific-Basin Finance Journal. 2021; 68: 101613. doi: 10.1016/j.pacfin.2021.101613

[11] Houston JF, Shan H. Corporate ESG profiles and banking relationships. The Review of Financial Studies. 2022; 35(7): 3373–3417. doi: 10.1093/rfs/hhab125

[12] Fang M, Nie H, Shen X. Can enterprise digitization improve ESG performance?. Economic Modelling. 2023; 118: 106101. doi: 10.1016/j.econmod.2022.106101

[13] Jang GY, Kang HG, Kim W. Corporate executives’ incentives and ESG performance. Finance Research Letters. 2022; 49: 103187. doi: 10.1016/j.frl.2022.103187

[14] Wang Y, Liu X, Wan D. Stock market openness and ESG performance: Evidence from Shanghai-Hong Kong connect program. Economic Analysis and Policy. 2023; 78: 1306–1319. doi: 10.1016/j.eap.2023.05.005

[15] McGuinness PB, Vieito JP, Wang M. The role of board gender and foreign ownership in the CSR performance of Chinese listed firms. Journal of Corporate Finance. 2017; 42: 75–99. doi: 10.1016/j.jcorpfin.2016.11.001

[16] Bai F, Shang M, Huang Y. Corporate culture and ESG performance: Empirical evidence from China. Journal of Cleaner Production. 2024; 437: 140732. doi: 10.1016/j.jclepro.2024.140732

[17] Barros V, Matos PV, Sarmento JM, Vieira PR. M&A activity as a driver for better ESG performance. Technological Forecasting and Social Change. 2022; 175: 121338. doi: 10.1016/j.techfore.2021.121338

[18] Li BH, Hou BC, Yu WT, Lu XB, Yang CW. Applications of artificial intelligence in intelligent manufacturing: a review. Frontiers of Information Technology & Electronic Engineering. 2017; 18(1): 86–96. doi: 10.1631/fitee.1601885

[19] Xue Q, Jin Y, Zhang C, Xue Q, Jin Y, Zhang C. ESG rating results and corporate total factor productivity. International Review of Financial Analysis. 2024; 103381. doi: 10.1016/j.irfa.2024.103381

[20] Yu X, Chen Y. Does ESG advantage promote total factor productivity (TFP)? Empirical evidence from China’s listed enterprises. Applied Economics. 2024; 1–17. doi: 10.1080/00036846.2024.2336886

[21] Wen H, Zhao Z. How does China’s industrial policy affect firms’ R&D investment? Evidence from ‘Made in China 2025’. Applied Economics. 2021; 53(55): 6333–6347. doi: 10.1080/00036846.2020.1717429

[22] Liu X S, Megginson W L, Xia J. Industrial policy and asset prices: Evidence from the Made in China 2025 policy. Journal of Banking & Finance. 2022; 142: 106554. doi: 10.1016/j.jbankfin.2022.106554

[23] Bennett B, Stulz R, Wang Z. Does the stock market make firms more productive?. Journal of Financial Economics. 2020; 136(2): 281–306. doi: 10.1016/j.jfineco.2019.09.006

[24] Bender S, Bloom N, Card D, Van Reenen J, Wolter S. Management practices, workforce selection, and productivity. Journal of Labor Economics. 2018; 36(S1): S371–S409. doi: 10.1086/694107

[25] De PK, Nagaraj P. Productivity and firm size in India. Small Business Economics. 2014; 42: 891–907. doi: 10.1007/s11187-013-9504-x

[26] Müller JM, Buliga O, Voigt KI. Fortune favors the prepared: How SMEs approach business model innovations in Industry 4.0. Technological Forecasting and Social Change. 2018; 132: 2–17. doi: 10.1016/j.techfore.2017.12.019

[27] Ding X, Sheng Z, Appolloni A, Shahzad M, Han S. Digital transformation, ESG practice, and total factor productivity. Business Strategy and the Environment. 2024; 33(5): 4547–4561. doi: 10.1002/bse.3718

[28] Yang J, Ying L, Gao M. The influence of intelligent manufacturing on financial performance and innovation performance: the case of China. Enterprise Information Systems. 2020; 14(6): 812–832. doi: 10.1080/17517575.2020.1746407

[29] Ying L, Liu X, Li M, Sun L, Xiu P, Yang J. How does intelligent manufacturing affects enterprise innovation? The mediating role of organisational learning. Enterprise Information Systems. 2022; 16(4): 630–667. doi: 10.1080/17517575.2021.1939424

[30] Brynjolfsson E, Collis A. How should we measure the digital economy. Harvard Business Review. 2019; 97(6): 140–148.

[31] Li G, Branstetter LG. Does “Made in China 2025” work for China? Evidence from Chinese listed firms. Research Policy. 2024; 53(6): 105009. doi: 10.1016/j.respol.2024.105009

[32] Acemoglu D, Restrepo P. Automation and new tasks: How technology displaces and reinstates labor. Journal of Economic Perspectives. 2019; 33(2): 3–30. doi: 10.1257/jep.33.2.3

[33] Harrison A, Rodríguez-Clare A. Trade, foreign investment, and industrial policy for developing countries. Handbook of Development Economics. 2010; 5: 4039–4214. doi: 10.1016/B978-0-444-52944-2.00001-X

[34] Sun L, Saat NAM. How does intelligent manufacturing affect the ESG performance of manufacturing firms? Evidence from China. Sustainability. 2023; 15(4): 2898. doi: 10.3390/su15042898

[35] Bajari P, Chernozhukov V, Hortaçsu A, Suzuki J. The impact of big data on firm performance: An empirical investigation. AEA Papers and Proceedings. 2019; 109: 33–37. doi: 10.1257/pandp.20191000

[36] Subramaniam M, Youndt MA. The influence of intellectual capital on the types of innovative capabilities. Academy of Management Journal. 2005; 48(3): 450–463. doi: 10.5465/AMJ.2005.17407911

[37] Yu FF. Analyst coverage and earnings management. Journal of Financial Economics. 2008; 88(2): 245–271. doi: 10.1016/j.jfineco.2007.05.008

[38] Graetz G, Michaels G. Robots at work. Review of Economics and Statistics. 2018; 100(5): 753–768. doi: 10.1016/s0140-6736(06)69092-2

[39] Liang Y, Cai C, Huang Y. The effect of corporate social responsibility on productivity: Firm-level evidence from Chinese listed companies. Emerging Markets Finance and Trade. 2022; 58(12): 3589–3607. doi: 10.1080/1540496X.2020.1788537

[40] Deng X, Li W, Ren X. More sustainable, more productive: Evidence from ESG ratings and total factor productivity among listed Chinese firms. Finance Research Letters. 2023; 51: 103439. doi: 10.1016/j.frl.2022.103439

[41] Flammer C. Does product market competition foster corporate social responsibility? Evidence from trade liberalization. Strategic Management Journal. 2015; 36(10): 1469–1485. doi: 10.1002/smj.2307

[42] Jones DA, Willness CR, Madey S. Why are job seekers attracted by corporate social performance? Experimental and field tests of three signal-based mechanisms. Academy of Management Journal. 2014; 57(2): 383–404. doi: 10.5465/amj.2011.0848

[43] Eliwa Y, Aboud A, Saleh A. ESG practices and the cost of debt: Evidence from EU countries. Critical Perspectives on Accounting. 2021; 79: 102097. doi: 10.1016/j.cpa.2019.102097

[44] Apergis N, Poufinas T, Antonopoulos A. ESG scores and cost of debt. Energy Economics. 2022; 112: 106186. doi: 10.1016/j.eneco.2022.106186

[45] Levinsohn J, Petrin A. Estimating production functions using inputs to control for unobservables. The Review of Economic Studies. 2003; 70(2): 317–341. doi: 10.1111/1467-937x.00246

[46] Olley GS, Pakes A. The dynamics of productivity in the telecommunications equipment industry. Econometrica. 1996; 64(6): 1263–1297. doi: 10.2307/2171831

[47] Wooldridge JM. On estimating firm-level production functions using proxy variables to control for unobservables. Economics Letters. 2009; 104(3): 112–114. doi: 10.1016/j.econlet.2009.04.026

[48] Hadlock CJ, Pierce JR. New evidence on measuring financial constraints: Moving beyond the KZ index. The Review of Financial Studies. 2010; 23(5): 1909–1940. doi: 10.1093/rfs/hhq009

[49] Quan X, Li C. Cost stickiness mitigation effect of intelligent manufacturing: On a quasi-natural experiment of Chinese intelligent manufacturing demonstration project. Economic Research Journal (Chinese). 2022; 57(04): 68–84. https://mall.cnki.net/magazine/Article/JJYJ202204006.htm

[50] Huang Q, Yu Y, Zhang S. Internet development and productivity growth in manufacturing industry: Internal mechanism and China experiences. China Industrial Economics (Chinese). 2019; 8: 5–23. https://mall.cnki.net/magazine/Article/GGYY201908001.htm

[51] Zhang L, Tao Y, Nie C. Does broadband infrastructure boost firm productivity? Evidence from a quasi-natural experiment in China. Finance Research Letters. 2022; 48: 102886. doi: 10.1016/j.frl.2022.102886

[52] Ghisetti C, Montresor S, Vezzani A. Design and environmental technologies: Does ‘green-matching’actually help?. Research Policy. 2021; 50(5): 104208. doi: 10.1016/j.respol.2021.104208

[53] Irani RM, Oesch D. Monitoring and corporate disclosure: Evidence from a natural experiment. Journal of Financial Economics. 2013; 109(2): 398–418. doi: 10.1016/j.jfineco.2013.02.021

[54] Hu M, Xiong W, Xu C. Analyst coverage, corporate social responsibility, and firm value: Evidence from China. Global Finance Journal. 2021; 50: 100671. doi: 10.1016/j.gfj.2021.100671

[55] Huang B, Li HT, Liu JQ, Lei J. Digital technology innovation and the high-quality development of Chinese enterprises: Evidence from enterprise’s digital patents. Economic Research Journal (Chinese). 2023; 58(03): 97–115. https://mall.cnki.net/magazine/Article/JJYJ202303006.htm

[56] Acemoglu D, Restrepo P. The race between man and machine: Implications of technology for growth, factor shares, and employment. American Economic Review. 2018; 108(6): 1488–1542. doi: 10.1257/aer.20160696

[57] Chen N, Sun D, Chen J. Digital transformation, labour share, and industrial heterogeneity. Journal of Innovation & Knowledge. 2022; 7(2): 100173. doi: 10.1016/j.jik.2022.100173

[58] Hong L, Liu X, Zhan H, Han F. Use of industrial robots and Chinese enterprises’ export quality upgrading: Evidence from China. The Journal of International Trade & Economic Development. 2022; 31(6): 860–875. doi: 10.1080/09638199.2021.2018021

[59] Kama I. On the market reaction to revenue and earnings surprises. Journal of Business Finance & Accounting. 2009; 36(1‐2): 31–50. doi: 10.1111/j.1468-5957.2008.02121.x

[60] Stel AV, Carree M, Thurik R. The effect of entrepreneurial activity on national economic growth. Small Business Economics. 2005; 24: 311–321. doi: 10.1007/s11187-005-1996-6

